# Determinants of adverse birth outcomes among women delivered in public hospitals of Ethiopia, 2020

**DOI:** 10.1186/s13690-021-00776-0

**Published:** 2022-01-04

**Authors:** Muktar Abadiga, Getu Mosisa, Reta Tsegaye, Adugna Oluma, Eba Abdisa, Tilahun Bekele

**Affiliations:** School of Nursing and midwifery, Institute of Health Sciences, Wollega University, Nekemte, Ethiopia

**Keywords:** Determinant, Adverse birth outcome, Mothers, Western Ethiopia

## Abstract

**Background:**

Adverse birth outcome is a common health problem consisting of several health effects involving pregnancy and the newborn infant. Infants with one or more adverse birth outcomes are at greater risk for mortality and a variety of health and developmental problems. Factors such as the age of the mother, antepartum hemorrhage, history of abortion, gestational age, anemia, and maternal undernutrition have predisposed the mother to adverse birth outcome. For appropriate prevention of the adverse birth outcomes, data pertaining to determinants of adverse birth outcomes are important. Therefore, this study was aimed to assess the determinants of adverse birth outcomes among women who give birth in public hospitals of western Ethiopia.

**Methods:**

An institutional-based unmatched prospective case-control study was conducted from February 15 to April 15, 2020, in selected public hospitals of western Ethiopia. From mothers who gave birth in public hospitals of Wollega zones, 165 cases and 330 controls were selected. Mothers with adverse birth outcomes were cases and mothers without adverse birth were controls. Data was collected by structured interviewer-administered questionnaires. In addition to the interview, the data collectors abstracted clinical data by reviewing the mother and the babies’ medical records. The collected data were entered into Epi info version 7 and exported to SPSS version 21 for analysis. Finally, multivariable logistic regression was used to identify determinants of adverse birth outcomes at *P*-value < 0.05.

**Results:**

A total of 495 mothers (165 cases and 330 controls) were included in the study with a mean age of 28.48 + 5.908. Low ANC visit (AOR = 3.92: 95% CI; 1.86, 8.2), premature rupture of membrane (AOR = 2.83: 95% CI; 1.72,4.64), being Anemic (AOR = 2: 95% CI; 1.16,3.44), pregnancy induced-hypertension (AOR = 2.3:95% CI; 1.4,3.85), not getting dietary supplementation (AOR = 2.47:95% CI; 1.6,3.82), and physical abuse (AOR = 2.13: 95% CI; 1.05,4.32) were significantly associated with the development of the adverse birth outcome.

**Conclusion:**

Low antenatal care visit, being anemic, premature rupture of membrane, pregnancy-induced hypertension, not getting dietary supplementation, and physical abuse were determinants of adverse birth outcomes. The clinicians should play a pivotal role to improve antenatal care follow up, counsel, and supplement recommended diets and minimize violence and abuse during pregnancy.

**Supplementary Information:**

The online version contains supplementary material available at 10.1186/s13690-021-00776-0.

## Plain English Summary

The adverse birth outcome is a common health problem consisting of several health effects involving pregnancy and the newborn infant. Birth outcomes are measures of health at birth and their magnitude is dramatically decreased in the past 40 years. However; there is still a large gap between developing and developed countries. Infants with one or more adverse birth outcomes are at greater risk for mortality and a variety of health and developmental problems. For appropriate prevention of adverse birth outcomes, data pertaining to determinants of adverse birth outcomes are important. An institutional-based unmatched prospective case-control study was conducted from February 15 to April 15, 2020, in selected public hospitals of western Ethiopia. In this study, low Antenatal care (ANC) visits, being anemic, premature rupture of membrane, pregnancy-induced hypertension, not getting dietary supplementation, and physical abuse were determinants of adverse birth outcomes. The clinicians should play a pivotal role to improve ANC follow up, counsel, and supplement recommended diets and minimize violence and abuse during pregnancy.

**Supplementary Information:**

The online version contains supplementary material available at 10.1186/s13690-021-00776-0.

## Background

Although most pregnancy and childbirth are a joyful experience for most women, it sometimes ends up in adverse birth outcome [1]. The adverse birth outcome is a multifactorial outcome that mainly includes preterm birth, low birth weight, stillbirth, macrosomia, congenital anomaly, and infant/neonatal death. It is a common health problem consisting of a several health effects involving pregnancy and the newborn infant [2]. Preterm birth is a live birth before 37 completed weeks of gestation and is the leading cause of neonatal mortality [3]. Low birthweight is infants who weigh less than 2500 g (about 5 ½ pounds) at birth [4]. On the other hand, stillbirth is when the infant died in the womb after 28 weeks of gestation [5].

Birth outcomes are measures of health at birth and their magnitude is dramatically decreased in the past 40 years. However; there is still a large gap between developing and developed countries [2]. Globally, about 15 million babies are born prematurely each year, from which Sub-Saharan Africa and South Asia account for more than 60% [6, 7]. Of all births occurred worldwide, about 20 million are low birth weight and 15% of this occurs in sub-Saharan Africa [4]. Worldwide, nearly 3 million stillbirths occur annually, 98% of which occur in developing countries [8]. On the other hand, more than 45% of under-fives death occur within the first 28 days of life [9]. The overall prevalence of adverse birth outcomes is 25.7% in rural India [10], 15.61% in Zimbabwe [11], and 10.8% in rural Uganda [12]. In Ethiopia, the prevalence of adverse birth outcome is 18.3% in Hawassa town [13], 23% in Gondor university hospital [14], 32.5% in Dessie referral hospital [15], 18.2% in Butajira general hospital [16], and 31.8% in North Wollo zone [17].

Infants with one or more adverse birth outcomes are at greater risk for mortality and a variety of health and developmental problems [7]. Babies with adverse birth outcomes are more likely to experience complications like respiratory, immunologic, nervous system, and behavioral problems [7, 18]. Preterm birth and low birth weight are critical determinants of child survival, disabilities, stunting, and long-term adverse consequences [19]. Low birth weight infants may suffer the risk of developing many complications such as heart problems, anemia, chronic lung disorders, growth retardation, and inhibited cognitive developments [20]. Similarly, preterm birth imposes infants to physical and neurological problems which may lead them to lifelong disabilities [21]. Preterm complications are also the leading cause of approximately 27% of neonatal deaths which accounts for about four million every year [22].

The etiologies of adverse birth outcomes are multifactorial and not completely understood yet. Studies have shown that sociodemographic factors such as the age of the mother [12, 13, 16, 17, 23, 24], educational level [23], level of income [25, 26], and place of residence [16, 17, 26] showed association with adverse birth outcome. Obstetric factors such as the previous history of adverse birth outcome [13, 14], antepartum hemorrhage [14], history of abortion [16], gestational age [11], pregnancy interval [27], obstetric complications [11, 15–17] and the number of parities [12, 17] were associated with adverse birth outcome. Other factors such as the presence of chronic diseases [13], hypertensive disorder during pregnancy [14], anemia [15, 27], no dietary counseling [17, 27], low antenatal care visit [12–15, 17, 27], substance use [25] and malaria [28] were also associated with adverse birth outcome. Factors associated with adverse birth outcomes are not the same across different cultures and socioeconomic statuses within a society.

For appropriate planning and implementing proper interventions to prevent the adverse birth outcome, data pertaining to determinants of adverse birth outcome are very important. Although there are studies on the various forms of adverse birth outcomes, there is limited data on the overall adverse birth outcomes in Ethiopia. To the best of our knowledge, this is the first study to assess adverse birth outcomes and their associated factors in the western part of Ethiopia. Therefore, this study was aimed to assess the determinants of adverse birth outcomes among women who gave birth in public hospitals of western Ethiopia. The finding of this study will help the health care providers in planning health interventions to improve the wellbeing of children and women.

## Methods

### Study design, setting and population

An institutional-based unmatched prospective case-control study was conducted from February 15 to April 15, 2020, in selected public hospitals of Wollega zones, western Ethiopia. According to the 2010 Central Statistical Agency (CSA) report, the total population of Wollega zone is 3,345,675 (52% females and 48% males) [29]. The Wollega zone is administratively divided into 4 zones, namely, the East Wollega zone, Horro Guduru Wollega zone, West Wollega zone, and Kellem Wollega zone. In the Wollega zone, there are 2 comprehensive specialized hospitals, 9 general hospitals, 13 primary hospitals, 102 health centers, and 401 health posts. This study was conducted in six randomly selected public hospitals found in Wollega zones, namely; Nekemte specialized hospital, Arjo hospital, Wollega university specialized hospital, Shambu general hospital, Gimbi general hospital, and Nedjo general hospital. An estimated total number of 85,345 births have been registered annually in the zones. The source population was all mothers who gave birth in public hospitals of the four Wollega zones. The study population was all mothers who give birth at the six selected public hospitals of Wollega zones during the study period.

All mothers who gave birth at the selected hospitals of Wollega zones were included in the study. Women with induced termination of pregnancy for medical reasons, women with a serious general medical condition, unknown last menstrual period (LMP) or not reliable ultrasonography and unable to communicate were excluded. Cases were mothers with at least one adverse birth outcome (preterm birth; < 37 gestational weeks at birth, low birth weight; the weight of < 2.5 kg at birth, stillbirth; infant died in the womb or during the intrapartum period after 28 weeks of gestation, macrosomia; birth-weight over 4000 g irrespective of gestational age, birth defect/congenital anomaly; structural changes in one or more parts of the infant’s body that are present at birth, neonatal death; the death of infant or neonates within 28 days of life, small for gestational age; birth weight below the 10th percentile for the gestational age). Controls were mothers without adverse birth outcomes (≥ 37 gestational weeks at birth, live birth, weight ≥ 2.5 kg at birth and not greater than 4 kg, no congenital anomaly, the appropriate size for gestational age, and no neonatal death).

### Sample size determination and sampling techniques

The Sample size was calculated using the double population proportion using EPI-Info 3.5.1 statistical software. The proportion of low educational status, advanced maternal age, rural residence, age at first sex ≤14, and the age difference between the partner at first intercourse ≥5 years was used to determine the sample size from a study done in Debre Tabor town [23]. Maternal age 45–54 years was chosen as an independent variable since it brought a higher sample size among other computed explanatory variables. The assumptions for the sample size calculation were as follows: minimum detectable odds ratio of 2, a confidence level of 95% (Zα/2 = 1.96), power of 80% (Zβ = 0.80), a case to control ratio of 1:2, and proportion of case among an exposed group of 22.0% [23]. After adding a 10% non-response rate, the total calculated sample size was 543 (181 cases and 362 controls).

Eligible cases were selected consecutively and the consecutive two controls were selected until the required sample size was achieved. The number of cases and controls were proportionally allocated to each hospital based on the number of mothers who gave birth at each of the selected hospitals in 4 months before the data collection time. Then, the average number of mothers expected to give birth per 2 months in each of the respective hospitals was estimated. Accordingly, we included 42 cases and 84 controls in Nekemte specialized Hospital, 38 cases and 76 controls in Wollega university specialized hospital, 27 cases and 54 controls in Gimbi general Hospital, 22 cases and 44 controls in Shambu general hospital, 29 cases and 58 controls in Nedjo hospital and 23 cases and 46 controls in Arjo Hospital.

### Data collection tool and procedure

Data was collected by structured interviewer-administered questionnaire adapted from the Ethiopian Demographic and Health Survey (EDHS) and other similar studies [11–14, 16, 17, 23, 27, 30]. The outcome variable was the adverse birth outcome and the independent variables were socio-demographic variables, obstetric related factors, pre-conception related factors, nutritional and dietary related factors, behavior-related factors, mothers’ history of pre-existing medical illness, and health facility-related factors. The interview was held in a separate room after a woman is stabilized and ready to be discharged. In addition to the interview, the data collectors abstracted clinical data by reviewing the mother and the babies’ medical records. Anthropometric measurements were also made for both the mothers and the newborns. The baby’s weight was measured by the data collectors within 15 min after delivery using a calibrated digital scale and rounded to the nearest 0.1 g. Mothers’ mid-upper arm circumference was measured using flexible non-stretchable tape measure. Maternal hemoglobin level was reviewed from mothers’ cards to determine anemia. The data was collected by 12 trained BSc midwives recruited from other hospitals not included in this study for 2 months. Six Master of Science qualified midwives supervised the overall data collection process.

### Data quality control

The questionnaire was translated to the local language Afan Oromo and then back to English by two different language experts to check for consistency. Five percent of the questionnaire (27 participants) was pre-tested at the same study area 5 days before data collection and modification was made based on the pre-test result. The ratio of a case to control used in the pretest was 1:2 (9 cases and 18 controls). Five days of training on the objectives of the study, sampling technique, ethical consideration, and data collection techniques was given for data collectors and supervisor. Continuous follow-up and supervision of data collection were made by the supervisors. The collected data were checked by the supervisor daily for completeness.

### Data processing and analysis

The collected data were coded, cleaned, and entered into Epi info version 7 and exported to SPSS version 21 for analysis. Descriptive statistics like frequencies and percentages were performed as univariate analysis. In this study, cases were coded as 1, and controls were coded as 0 for analysis. Bivariable logistic regression analysis was used to assess the unadjusted effect of each independent variable on the dependent variable. Variables that have a *P*-value less than 0.25 were entered into a multivariable logistic regression model. A multivariable logistic regression model was fitted to identify independent determinants of adverse birth outcomes. Model fitness was tested with Hosmer-Lemeshow goodness of fit test and omnibus tests of model coefficients. Variance inflation factor (VIF) and tolerance test were also used to check multicollinearity. Adjusted odds ratio (AOR) with a 95% confidence interval (CI) was calculated to determine the strength of an association. *P*-value < 0.05 was considered statistically significant in multivariable logistic regression.

### Operational definitions

Preterm birth: is when a baby is born before 37 completed weeks of gestation but after 28 weeks of gestation. Low birth weight: is when a baby’s birth weight is below 2500 g or below 2.5 kg. Macrosomia: is defined as birth-weight over 4 kg irrespective of gestational age. Stillbirth: is when the infant died in the womb or during the intrapartum period after 28 weeks of gestation. Birth defect/congenital anomaly: is when there are structural changes in one or more parts of the infant’s body that are present at birth. Adverse birth outcome: is when a woman met at least one of the above conditions. No adverse birth outcome: Those women who gave normal live birth without the above-mentioned abnormal birth outcome [5]. Perceived stress: A mother is considered as stressed if perceived stress score is above mean and not stressed if below calculated mean value of scores. Danger sign of pregnancy: A mother is considered as having danger sign if she developed at least one from nine danger signs stated in the world health organization (WHO) guide list and no danger sign if no any sign developed.

## Results

### Socio-demographic characteristics of the respondents

From the total of 543 eligible participants (181 cases and 362 controls), 495 mothers (165 cases and 330 controls) were participated in the study making a response rate of 91.2%. The mother’s age was lying between 15 and 44 years with a mean of 28.48 + 5.908 SD. The majority of the study participants, 141 (85.45%) of the cases and 286 (86.67%) of the controls were Oromo. Regarding the religion of the respondents, 86 (52.12%) of the cases, and 160 (48.48%) of the controls were protestant. About 153 (92.73%) of the cases and 311 (94.24%) of the controls were married. Regarding educational status, 47 (28.48%) of the cases and 108 (32.7%) of controls had a diploma and above. The majority of the study participants, 104 (63.03%) of the cases and 212 (64.24%) of controls were from rural. More than half of the study participants, 105 (63.64%) of the cases and 189 (57.27%) of the controls were married at an age between 18 and 23 years. Regarding monthly income, 37(22.42%) of the cases and 75 (22.73%) of the controls get a monthly income of 2001–3000 Ethiopian birr. Concerning family size, 107 (64.85%) of the cases, and 197(59.7%) of the controls have 4–6 family size (Table 1).

**Table 1:** Socio-demographic characteristics of mothers attending birth at public hospitals of Wollega zones, 2020 (*n* = 495; Cases: 165 and controls: 330)

Variables	Control n (%)	Cases n (%)	Total n (%)
**Ethnicity of mothers**			
Oromo	286 (86.67)	141 (85.45)	427 (86.3)
Amhara	35 (10.6)	21 (12.73)	56 (11.3)
Gurage	9 (2.73)	3 (1.82)	12 (2.4)
**Religion of mothers**			
Protestant	160 (48.48)	86 (52.12)	246 (49.7)
Orthodox	86 (26.06)	39 (23.64)	125 (25.3)
Catholic	13 (3.94)	8 (4.85)	21 (4.2)
Muslim	62 (18.78)	29 (17.58)	91 (18.4)
Others	9 (2.73)	3 (1.82)	12 (2.4)
**Marital status**			
Married	311 (94.24)	153 (92.73)	464 (93.7)
Single	6 (1.82)	3 (1.82)	9 (1.8)
Divorced	9 (2.73)	6 (3.64)	15 (3)
Widowed	4 (1.21)	3 (1.82)	7 (1.4)
**Educational status**			
Unable to read and write	72 (21.8)	40 (24.24)	112 (22.6)
Completed grade 1–8	84 (25.5)	42 (25.45)	126 (25.5)
Completed grade 9–12	66 (20)	36 (21.82)	102 (20.6)
Diploma and above	108 (32.7)	47 (28.48)	155 (31.3)
**Residence**			
Rural	212 (64.24)	104 (63.03)	316 (63.8)
Urban	118 (35.76)	61 (36.97)	179 (36.2)
**Age category**			
15–24	89 (26.97)	43 (26.06)	132 (26.7)
25–34	176 (53.33)	93 (56.36)	269 (54.3)
35–44	65 (19.7)	29 (17.58)	94 (19)
**Age at 1st marriage**			
< 18 years	110 (33.33)	51 (30.91)	161 (32.5)
18–23 years	189 (57.27)	105 (63.64)	294 (59.4)
> 23 years	31 (9.39)	9 (5.45)	40 (8.1)
**Monthly income**			
< 1000 birr	67 (20.3)	40 (24.24)	107 (21.6)
1000–2000 birr	54 (16.36)	31 (18.79)	85 (17.2)
2001–3000 birr	75 (22.73)	37 (22.42)	112 (22.6)
3001–4000 birr	59 (17.88)	28 (16.97)	87 (17.6)
> 4000	75 (22.73)	29 (17.58)	104 (21)
**Family size**			
1–3	98 (29.7)	37 (22.42)	135 (27.3)
4–6	197 (59.7)	107 (64.85)	304 (61.4)
> 6	35 (10.7)	21 (12.73)	56 (11.3)

### Obstetrics related characteristics of the respondents

Regarding the number of births, 106 (64.24%) of the cases and 217 (65.76%) of the controls have less than 3 births. The birth space for 54 (32.73%) cases and 121 (36.67%) of controls were more than 23 months. The majority of the study participants, 118 (71.52%) of the cases 247 (74.85%) of the controls were used family planning before the current pregnancy. The majority of participants, 125 (75.76%) of the cases and 260 (78.79%) of the controls were planned their pregnancy. About 60 (36.4%) of the cases and 182 (55.15%) of the controls were attended ANC more than four times. Concerning danger signs, 123 (74.55%) of the cases and 267 (80.9%) of the controls were didn’t experienced danger signs during pregnancy. More than half, 88 (53.33%) of the cases and 196 (59.39%) of the controls were delivered through SVD. Concerning the previous history of adverse birth outcomes, 131 (79.4%) of the cases, and 271 (82.12%) of the controls had no history of adverse birth outcomes. About 106 (64.24%) of the cases and 280 (84.85%) of the controls didn’t experience PROM, and most of the labor started spontaneously for 125 (75.76%) of cases and 269 (81.52%) of controls (Table 2).

**Table 2:** Obstetrics related characteristics of mothers attending birth at public hospitals of Wollega zones, 2020 (*n* = 495; Cases: 165 and controls: 330)

Variables	Control n (%)	Cases n (%)	Total n (%)
**Number of births**			
< 3	217 (65.76)	106 (64.24)	323 (65.3)
3–5	81 (24.55)	47 (28.48)	128 (25.9)
> 5	32 (9.70)	12 (7.27)	44 (8.9)
**Duration between births**			
1st pregnancy	79 (23.94)	49 (29.70)	128 (25.9)
< 12 months	16 (4.85)	10 (6.06)	26 (5.3)
12–17 months	26 (7.88)	10 (6.06)	36 (7.3)
18–23 months	88 (26.67)	42 (25.45)	130 (26.3)
> 23 months	121 (36.67)	54 (32.73)	175 (35.4)
**Family planning utilization**			
Ye	247 (74.85)	118 (71.52)	365 (73.7)
No	83 (25.15)	47 (28.48)	130 (26.3)
**Plan of pregnancy**			
Yes	260 (78.79)	125 (75.76)	385 (77.8)
No	70 (21.21)	40 (24.24)	110 (22.2)
**Antenatal care attendance**			
One time	16 (4.85)	33 (20)	49 (9.9)
Two times	46 (13.94)	33 (20)	79 (16)
Three times	86 (26.06)	39 (23.6)	125 (25.3)
> =4 times	182 (55.15)	60 (36.4)	242 (48.9)
**Danger sign of pregnancy**			
Yes	63 (19.1)	42 (25.45)	105 (21.2)
No	267 (80.9)	123 (74.55)	390 (78.8)
**Mode of delivery**			
Spontaneous vaginal delivery	196 (59.39)	88 (53.33)	284 (57.4)
Forceps	18 (5.45)	10 (6.06)	28 (5.7)
Cesarean section	66 (20)	43 (26.06)	109 (22)
Vacuum delivery	45 (13.64)	20 (12.12)	65 (13.1)
Destructive delivery	5 (1.52)	4 (2.42)	9 (1.8)
**History of adverse birth outcome**			
Yes	59 (17.88)	34 (20.6)	93 (18.8)
No	271 (82.12)	131 (79.4)	402 (81.2)
**Premature rupture of membrane**			
Yes	50 (15.15)	59 (35.76)	109 (22)
No	280 (84.85)	106 (64.24)	386 (78)
**Labor started**			
Spontaneously	269 (81.52)	125 (75.76)	394 (79.6)
Induced	61 (18.48)	40 (24.24)	101 (20.4)

### Mothers medical history and health facility related characteristics of respondents

The majority of the participants, 288 (87.27%) of the controls and 124 (75.15%) of the cases and nearly all, 322 (97.58%) of the controls and 160 (96.97%) of the cases had no history of hypertension and cardiac diseases respectively. Three hundred eleven (94.24%) of the controls and 154 (93.33%) of the cases were HIV negative. The majority of participants, 291 (88.18%) of the controls and 120 (72.73%) of the cases, 317 (96.06%) of the controls and 154 (93.33%) of the cases, and 289 (87.58%) of the controls and 154 (93.3%) of the cases had no history of Anemia, malaria, and STI respectively.

Concerning pregestational BMI, 258 (78.18%) of the controls, and 122 (73.94%) of the cases had 18–24 kg/m^2^. The majority of the participants, 281 (85.15%) of the controls and 108 (64.45%) of the cases had no pregnancy-induced hypertension. More than half, 192 (58.18%) of the controls and 111 (67.27%) of the cases had a height of < 150 cm. Regarding the time of ANC visit, 90 (54.55%) of the cases and 167 (50.6%) of the controls visited in the 2nd trimester. Almost all,321 (97.27%) of the controls and 163 (98.79%) of the cases delivered a single child. Labor duration has lasted for 8–15 h in 143 (43.33%) of the controls and 65 (39.4%) of the cases. The majority of participants, 217 (65.75%) of the controls and 102 (61.82%) of the cases travel less than 1 h to reach the health facility (Table 3).

**Table 3:** Mothers medical history and health facility related characteristics of mothers attending birth at public hospitals of Wollega zones, 2020 (*n* = 495; Cases: 165 and controls: 330)

Variables	Control n (%)	Cases n (%)	Total n (%)
**History of diabetes mellitus**			
Yes	21 (6.36)	10 (6.06)	31 (6.3)
No	309 (93.64)	155 (93.94)	464 (93.7)
**History of hypertension**			
Yes	42 (12.73)	41 (24.85)	83 (16.8)
No	288 (87.27)	124 (75.15)	412 (83.2)
**History of Cardiac disease**			
Yes	8 (2.42)	5 (3.03)	13 (2.6)
No	322 (97.58)	160 (96.97)	482 (97.4)
**Status of Human immunodeficiency virus status**			
Positive	9 (2.73)	4 (2.42)	13 (2.6)
Negative	311 (94.24)	154 (93.33)	465 (93.9)
Unknown	10 (3.03)	7 (4.24)	17 (3.4)
**Anemia**			
Yes	39 (11.82)	45 (27.27)	84 (17)
No	291 (88.18)	120 (72.73)	411 (83)
**Malaria**			
Yes	13 (3.94)	11 (6.67)	24 (4.8)
No	317 (96.06)	154 (93.33)	471 (95.2)
**Sexually transmitted infection**			
Yes	41 (12.42)	12 (7.27)	53 (10.7)
No	289 (87.58)	154 (93.3)	442 (89.3)
**Pregestational body mass index**			
< 18 kg/m2	35 (10.6)	17 (10.3)	52 (10.5)
18–24 kg/m2	258 (78.18)	122 (73.94)	380 (76.8)
> 24 kg/m2	37 (11.21)	26 (15.75)	63 (12.7)
**Pregnancy induced hypertension**			
Yes	49 (14.85)	57 (34.55)	106 (21.4)
No	281 (85.15)	108 (65.45)	389 (78.6)
**Maternal Height**			
> =150 cm	138 (41.82)	54 (32.73)	192 (38.8)
< 150 cm	192 (58.18)	111 (67.27)	303 (61.2)
**Dietary counselling**			
Yes	216 (65.45)	92 (55.76)	308 (62.2)
No	114 (34.55)	73 (44.24)	177 (37.8)
**Mid-upper arm circumference**			
< 23 cm	97 (29.4)	49 (29.7)	146 (29.5)
= > 23 cm	233 (70.6)	116 (70.3)	349 (70.5)
**Dietary supplementation**			
Yes	236 (71.52)	78 (47.27)	314 (63.4)
No	94 (28.48)	87 (52.73)	181 (36.6)
**Time of 1st antenatal care visit**			
1st Trimester	125 (37.88)	46 (27.88)	171 (34.5)
2nd trimester	167 (50.6)	90 (54.55)	257 (51.9)
3rd Trimester	38 (11.51)	29 (17.57)	67 (13.5)
**Number of children born**			
Multiple	9 (2.73)	2 (1.21)	11 (2.2)
Singleton	321 (97.27)	163 (98.79)	484 (97.8)
**Labour duration category**			
< 8 h	111 (33.64)	59 (35.76)	170 (34.3)
8-15 h	143 (43.33)	65 (39.4)	208 (42)
16-22 h	61 (18.48)	34 (20.6)	95 (19.2)
> 22 h	15 (4.55)	7 (4.24)	22 (4.4)
**Time to reach health facility**			
< 1 h	217 (65.75)	102 (61.82)	319 (64.4)
1-2 h	70 (21.21)	35 (21.21)	105 (21.2)
> 2 h	43 (13.03)	28 (16.97)	71 (14.3)

### Social and behavioral related characteristics of respondents

The majority of the participants, 281 (85.15%) of the controls and 128 (77.58%) of the cases had no history of substance use. Three hundred seven (93.03%) of the controls and 144 (87.27%) of the cases had no history of physical abuse and harassment. Almost all, 313 (94.85%) of the controls and 159 (96.36%) of the cases were not used traditional medicine, and about 254 (76.97%) of the controls and 113 (68.48%) of the cases didn’t experience stress during pregnancy (Table 4).

**Table 4:** Social and behavioral related characteristics of mothers attending birth at public hospitals of Wollega zones, 2020 (*n* = 495; Cases: 165 and controls: 330)

Variables	Control n (%)	Cases n (%)	Total n (%)
**History of substance abuse**			
Yes	49 (14.85)	37 (22.42)	86 (17.4)
No	281 (85.15)	128 (77.58)	409 (82.6)
**Physical abuse**			
Yes	23 (6.97)	21 (12.73)	44 (8.9)
No	307 (93.03)	144 (87.27)	451 (91.1)
**Use of traditional Medicine**			
Yes	17 (5.15)	6 (3.64)	23 (4.6)
No	313 (94.85)	159 (96.36)	472 (95.4)
**Experienced stress**			
Yes	76 (23.03)	52 (31.52)	128 (25.9)
No	254 (76.97)	113 (68.48)	367 (74.1)

### Bivariable logistic regression analysis of respondents

In the bivariable analysis, age at first marriage, income, family size, birth space, ANC attendance, danger sign of pregnancy, mode of delivery, premature rupture of the membrane, labor duration, history of hypertension, anemia, malaria, STI, pregnancy-induced hypertension, the height of mother, dietary counseling, dietary supplementation, time of ANC attendance, time to reach a health facility, history of substance abuse, physical abuse and stress were significantly associated with the adverse birth outcome at a *p*-value 0.25 (Table 5).

**Table 5:** Bivariable logistic regression analysis of adverse birth outcome among women gave birth at public hospitals of Wollega Zones, west Ethiopia, 2020 (*n* = 495; Cases: 165 and controls: 330)

Characteristics	Adverse birth outcome		COR (95%) CI	*P*- value
	Control n (%)	Cases n (%)		
**Ethnicity**				
Oromo	286 (86.67)	141 (85.45)	1	
Amhara	35 (10.6)	21 (12.73)	1.21 (0.68,2.17)	0.5
Gurage	9 (2.73)	3 (1.82)	0.67 (0.18,2.54)	0.56
**Religion of the mother**				
Protestant	160 (48.48)	86 (52.12)	1	
Orthodox	86 (26.06)	39 (23.64)	0.84 (0.53,1.34)	0.47
Catholic	13 (3.94)	8 (4.85)	1.14 (0.457,2.87)	0.77
Muslim	62 (18.78)	29 (17.58)	0.87 (0.52,1.45)	0.6
Others	9 (2.73)	3 (1.82)	0.62 (0.16,2.35)	0.48
**Marital status**				
Married	311 (94.24)	153 (92.73)	1	
Single	6 (1.82)	3 (1.82)	1.02 (0.25,4.12)	0.98
Divorced	9 (2.73)	6 (3.64)	1.35 (0.47,3.87)	0.57
Widowed	4 (1.21)	3 (1.82)	1.52 (0.34,6.89)	0.58
**Educational status**				
Unable to read and write	72 (21.8)	40 (24.24)	1.28 (0.76,2.14)	0.35
Completed grade 1–8	84 (25.5)	42 (25.45)	1.15 (0.69,1.9)	0.59
Completed grade 9–12	66 (20)	36 (21.82)	1.25 (0.74,2.13)	0.4
Diploma and above	108 (32.7)	47 (28.48)	1	
**Residence**				
Rural	212 (64.24)	104 (63.03)	1	
Urban	118 (35.76)	61 (36.97)	1.05 (0.7,1.55)	0.79
**Age category**				
15–24 years	89 (26.97)	43 (26.06)	1.08 (0.6,1.9)	0.78
25-34 years	176 (53.33)	93 (56.36)	1.18 (0.71,1.96)	0.5
35–44 years	65 (19.7)	29 (17.58)	1	
**Age at 1st marriage**				
< 18 years	110 (33.33)	51 (30.91)	1.6 (0.71,3.6)	0.26
18–23 years	189 (57.27)	105 (63.64)	1.9 (0.88,4.17)	0.1*
> 23 years	31 (9.39)	9 (5.45)	1	
**Monthly income**				
< 1000 birr	67 (20.3)	40 (24.24)	1.54 (0.86,2.76)	0.14*
1000–2000 birr	54 (16.36)	31 (18.79)	1.48 (0.8,2.75)	0.21*
2001–3000 birr	75 (22.73)	37 (22.42)	1.27 (0.7,2.28)	0.41
3001–4000 birr	59 (17.88)	28 (16.97)	1.23 (0.66,2.28)	0.52
> 4000 birr	75 (22.73)	29 (17.58)	1	
**Family size**				
1–3	98 (29.7)	37 (22.42)	1	
4–6	197 (59.7)	107 (64.85)	1.44 (0.92,2.25)	0.11*
> 6	35 (10.7)	21 (12.73)	1.59 (0.82,3.07)	0.17*
**Number of live births**				
< 3	217 (65.76)	106 (64.24)	1	
3–5	81 (24.55)	47 (28.48)	1.18 (0.77,1.82)	0.43
> 5	32 (9.70)	12 (7.27)	0.77 (0.38,1.55)	0.46
**Duration between pregnancy**				
1st pregnancy	79 (23.94)	49 (29.70)	1.39 (0.86,2.24)	0.18*
< 12 months	16 (4.85)	10 (6.06)	1.4 (0.6,3.28)	0.44
12–17 months	26 (7.88)	10 (6.06)	0.86 (0.39,1.9)	0.71
18–23 months	88 (26.67)	42 (25.45)	1.07 (0.657,1.74)	0.78
> 23 months	121 (36.67)	54 (32.73)	1	
**Family planning utilization**				
Yes	247 (74.85)	118 (71.52)	1	
No	83 (25.15)	47 (28.48)	1.18 (0.78,1.8)	0.427
**Plan of pregnancy**				
Yes	260 (78.79)	125 (75.76)	1	
No	70 (21.21)	40 (24.24)	1.19 (0.76,1.85)	0.44
**Antenatal care attendance**				
One time	16 (4.85)	33 (20)	6.25 (3.22,12.16)	< 0.001*
Two times	46 (13.94)	33 (20)	2.17 (1.27,3.71)	0.004*
Three times	86 (26.06)	39 (23.6)	1.37 (0.85,2.22)	0.19*
> =4 times	182 (55.15)	60 (36.4)	1	
**Danger sign of pregnancy**				
Yes	63 (19.1)	42 (25.45)	1.45 (0.93,2.26)	0.1*
No	267 (80.9)	123 (74.55)	1	
**Mode of delivery**				
Spontaneous vaginal delivery	196 (59.39)	88 (53.33)	1	
Forceps	18 (5.45)	10 (6.06)	1.24 (0.55,2.79)	0.61
Cesarean section	66 (20)	43 (26.06)	1.45 (0.92,2.29)	0.11*
Vacuum delivery	45 (13.64)	20 (12.12)	0.99 (0.55,1.77)	0.97
Destructive delivery	5 (1.52)	4 (2.42)	1.78 (0.47,6.79)	0.39
**History of adverse birth outcome**				
Yes	59 (17.88)	34 (20.6)	1.19 (0.745,1.91)	0.46
No	271 (82.12)	131 (79.4)	1	
**Premature rupture of membrane**				
Yes	50 (15.15)	59 (35.76)	3.12 (2.01,4.83)	< 0.001*
No	280 (84.85)	106 (64.24)	1	
**Labour started**				
Spontaneously	269 (81.52)	125 (75.76)	1	
Induced	61 (18.48)	40 (24.24)	1.41 (0.9,2.21)	0.135*
**History of diabetes mellitus**				
Yes	21 (6.36)	10 (6.06)	0.95 (0.44,2.07)	0.9
No	309 (93.64)	155 (93.94)	1	
**History of hypertension**				
Yes	42 (12.73)	41 (24.85)	2.27 (1.4,3.66)	0.001*
No	288 (87.27)	124 (75.15)	1	
**History of cardiac disease**				
Yes	8 (2.42)	5 (3.03)	1.26 (0.405,3.91)	0.69
No	322 (97.58)	160 (96.97)	1	
**Human immunodeficiency virus status**				
Positive	9 (2.73)	4 (2.42)	1	
Negative	311 (94.24)	154 (93.33)	1.1 (0.34,3.67)	0.86
Unknown	10 (3.03)	7 (4.24)	1.57 (0.34,7.22)	0.56
**Anemia**				
Yes	39 (11.82)	45 (27.27)	2.8 (1.73,4.5)	< 0.001*
No	291 (88.18)	120 (72.73)	1	
**Malaria**				
Yes	13 (3.94)	11 (6.67)	1.74 (0.76,3.97)	0.18*
No	317 (96.06)	154 (93.33)	1	
**Sexually transmitted infection**				
Yes	41 (12.42)	12 (7.27)	0.55 (0.28,1.08)	0.08*
No	289 (87.58)	154 (93.3)	1	
**Pregestational body mass index**				
< 18 kg/m2	35 (10.6)	17 (10.3)	0.97 (0.52,1.81)	0.93
18–24 kg/m2	258 (78.18)	122 (73.94)	1.45 (0.67,3.1)	0.34
> 24 kg/m2	37 (11.21)	26 (15.75)	1	
**Pregnancy induced hypertension**				
Yes	49 (14.85)	57 (34.55)	3.03 (1.95,4.7)	< 0.001*
No	281 (85.15)	108 (65.45)	1	
**Maternal height**				
> =150 cm	138 (41.82)	54 (32.73)	1	
< 150 cm	192 (58.18)	111 (67.27)	1.47 (0.99,2.18)	0.051*
**Dietary counselling**				
Yes	216 (65.45)	92 (55.76)	1	
No	114 (34.55)	73 (44.24)	1.5 (1.03,2.2)	0.036*
**Mid upper arm circumference**				
< 23 cm	97 (29.4)	49 (29.7)	1.01 (0.67,1.53)	0.94
= > 23 cm	233 (70.6)	116 (70.3)	1	
**Dietary supplementation**				
Yes	236 (71.52)	78 (47.27)	1	
No	94 (28.48)	87 (52.73)	2.8 (1.9,4.13)	< 0.001*
**Time of 1st antenatal care visit**				
1st Trimester	125 (37.88)	46 (27.88)	1	
2nd trimester	167 (50.6)	90 (54.55)	1.46 (0.96,2.24)	0.078*
3rd Trimester	38 (11.51)	29 (17.57)	2.07 (1.15,3.74)	0.015*
**Number of born child**				
Multiple	9 (2.73)	2 (1.21)	1	
Singleton	321 (97.27)	163 (98.79)	2.28 (0.48,10.7)	0.29
**Labor duration category**				
< 8 h	111 (33.64)	59 (35.76)	1	
8-15 h	143 (43.33)	65 (39.4)	0.85 (0.56,1.32)	0.47
16-22 h	61 (18.48)	34 (20.6)	1.05 (0.62,1.77)	0.86
> 22 h	15 (4.55)	7 (4.24)	0.87 (0.34,2.27)	0.79
**Time to reach health facility**				
< 1 h	217 (65.75)	102 (61.82)	0.72 (0.42,1.23)	0.23*
1-2 h	70 (21.21)	35 (21.21)	0.77 (0.41,1.43)	0.41
> 2 h	43 (13.03)	28 (16.97)	1	
**History of substance abuse**				
Yes	49 (14.85)	37 (22.42)	1.66 (1.03,2.67)	0.037*
No	281 (85.15)	128 (77.58)	1	
**Physical abuse**				
Yes	23 (6.97)	21 (12.73)	1.95 (1.04,3.63)	0.036*
No	307 (93.03)	144 (87.27)	1	
**Use of traditional medicine**				
Yes	17 (5.15)	6 (3.64)	0.69 (0.27,1.79)	0.45
No	313 (94.85)	159 (96.36)	1	
**Experienced stress**				
Yes	76 (23.03)	52 (31.52)	1.54 (1.01,2.33)	0.043*
No	254 (76.97)	113 (68.48)	1	

*COR* Crude Odd Ratio, *CI* Confidence Interval

*shows significant at *P*-value < 0.25

### Multivariable logistic regression analysis

In a multivariable logistic regression analysis, ANC attendance, PROM, being Anemic, PIHTN, dietary supplementation, and physical abuse/harassment were significantly associated with the adverse birth outcome. The odds of developing adverse birth outcomes were 3.92 times higher in mothers who had one-time ANC attendance than mothers who attended four and above times (AOR = 3.92: 95% CI; 1.86, 8.2). The odds of developing adverse birth outcomes were 2.83 times higher among mothers who developed PROM than mothers who do not develop (AOR = 2.83: 95% CI; 1.72,4.64). The odds of developing adverse birth outcomes were 2 times higher among anemic mothers than their counterparts (AOR = 2.0: 95% CI; 1.16,3.44). The study revealed that mothers who developed pregnancy-induced hypertension were 2.3 times higher odds of developing adverse birth outcomes than their counterparts (AOR = 2.3:95% CI;1.4,3.85). The odds of developing adverse birth outcomes were 2.47 times higher among mothers who didn’t get dietary supplementation than mothers who got dietary supplementation (AOR = 2.47:95% CI;1.6,3.82). Mothers who experienced physical abuse were 2.13 times more likely to develop adverse birth outcomes than mothers who do not exposed to physical abuse/harassment (AOR = 2.13: 95% CI;1.05,4.32) (Table 6).

**Table 6:** Multivariate logistic regression analysis of adverse birth outcome among mothers gave birth at public hospitals of Wollega Zones, west Ethiopia,2020 (*n* = 495; Cases: 165 and controls: 330)

Characteristics	Adverse birth outcome		COR (95%) CI	AOR (95%) CI	*P*-value
	Control n (%)	Cases n (%)			
**Age at 1st marriage**	110 (33.33)	51 (30.91)			
< 18 years	189 (57.27)	105 (63.64)	1.6 (0.71,3.6)	1.44 (0.57,3.68)	0.44
18–23 years	31 (9.39)	9 (5.45)	1.9 (0.88,4.17)	1.98 (0.83,4.7)	0.12
> 23 years	110 (33.33)	51 (30.91)	1	1	
**Monthly income**					
< 1000 birr	67 (20.3)	40 (24.24)	1.54 (0.86,2.76)	1.03 (0.5,2.09)	0.93
1000–2000 birr	54 (16.36)	31 (18.79)	1.48 (0.8,2.75)	1.48 (0.7,3.14)	0.31
2001–3000 birr	75 (22.73)	37 (22.42)	1.27 (0.7,2.28)	1.29 (0.64,2.59)	0.48
3001–4000 birr	59 (17.88)	28 (16.97)	1.23 (0.66,2.28)	1.24 (0.59,2.6)	0.56
> 4000 birr	75 (22.73)	29 (17.58)	1	1	
**Family size**					
1–3	98 (29.7)	37 (22.42)	1	1	
4–6	197 (59.7)	107 (64.85)	1.44 (0.92,2.25)	1.56 (0.87,2.82)	0.13
> 6	35 (10.7)	21 (12.73)	1.59 (0.82,3.07)	1.84 (0.79,4.3)	0.15
**Birth space**					
1st pregnancy	79 (23.94)	49 (29.70)	1.39 (0.86,2.24)	1.22 (0.68,2.17)	0.49
< 12 months	16 (4.85)	10 (6.06)	1.4 (0.6,3.28)	1.35 (0.47,3.85)	0.57
12–17 months	26 (7.88)	10 (6.06)	0.86 (0.39,1.9)	0.4 (0.16,1.0)	0.07
18–23 months	88 (26.67)	42 (25.45)	1.07 (0.657,1.74)	1.1 (0.64,1.96)	0.7
> 23 months	121 (36.67)	54 (32.73)	1		
**Antenatal care attendance**					
One time	16 (4.85)	33 (20)	6.25 (3.22,12.16)	3.92 (1.86,8.2)	< 0.001**
Two times	46 (13.94)	33 (20)	2.17 (1.27,3.71)	1.6 (0.9,2.9)	0.1
Three times	86 (26.06)	39 (23.6)	1.37 (0.85,2.22)	1.33 (0.78,2.26)	0.29
> =4 times	182 (55.15)	60 (36.4)	1	1	
**Danger sign of pregnancy**					
Yes	63 (19.1)	42 (25.45)	1.45 (0.93,2.26)	1.58 (0.94,2.65)	0.08
No	267 (80.9)	123 (74.55)	1	1	
**Mode of delivery**					
Spontaneous vaginal delivery	196 (59.39)	88 (53.33)	1	1	
Forceps	18 (5.45)	10 (6.06)	1.24 (0.55,2.79)	1.05 (0.35,3.1)	0.93
Cesarean section	66 (20)	43 (26.06)	1.45 (0.92,2.29)	1.21 (0.65,2.27	0.54
Vacuum delivery	45 (13.64)	20 (12.12)	0.99 (0.55,1.77)	1.1 (0.52,2.23)	0.83
Destructive delivery	5 (1.52)	4 (2.42)	1.78 (0.47,6.79)	1.27 (0.25,6.5)	0.77
**Premature rupture of membrane**					
Yes	50 (15.15)	59 (35.76)	3.12 (2.01,4.83)	2.83 (1.72,4.64)	< 0.001**
No	280 (84.85)	106 (64.24)	1	1	
**Labour started**					
Spontaneously	269 (81.52)	125 (75.76)	1	1	
Induced	61 (18.48)	40 (24.24)	1.41 (0.9,2.21)	0.94 (0.53,1.65)	0.82
**History of hypertension**					
Yes	42 (12.73)	41 (24.85)	2.27 (1.4,3.66)	1.16 (0.55,2.46)	0.69
No	288 (87.27)	124 (75.15)	1	1	
**Anemia**					
Yes	39 (11.82)	45 (27.27)	2.8 (1.73,4.5)	2.0 (1.16,3.44)	0.01**
No	291 (88.18)	120 (72.73)	1	1	
**Malaria**					
Yes	13 (3.94)	11 (6.67)	1.74 (0.76,3.97)	2.11 (0.76,5.83)	0.14
No	317 (96.06)	154 (93.33)	1	1	
**Sexually transmitted infection**					
Yes	41 (12.42)	12 (7.27)	0.55 (0.28,1.08)	0.55 (0.25,1.2)	0.13
No	289 (87.58)	154 (93.3)	1	1	
**Pregnancy induced hypertension**					
Yes	49 (14.85)	57 (34.55)	3.03 (1.95,4.7)	2.3 (1.4,3.85)	0.001**
No	281 (85.15)	108 (65.45)	1	1	
**Maternal height**					
> =150 cm	138 (41.82)	54 (32.73)	1	1	
< 150 cm	192 (58.18)	111 (67.27)	1.47 (0.99,2.18)	0.99 (0.587,1.67)	0.97
**Dietary counselling**					
Yes	216 (65.45)	92 (55.76)	1	1	
No	114 (34.55)	73 (44.24)	1.5 (1.03,2.2)	1.46 (0.94,2.27)	0.09
**Dietary supplementation**					
No	236 (71.52)	78 (47.27)	2.8 (1.9,4.13)	2.47 (1.6,3.82)	< 0.001**
Yes	94 (28.48)	87 (52.73)	1	1	
**Time of 1st antenatal care visit**					
1st Trimester	125 (37.88)	46 (27.88)	1	1	
2nd trimester	167 (50.6)	90 (54.55)	1.46 (0.96,2.24)	1.45 (0.89,2.37)	0.13
3rd Trimester	38 (11.51)	29 (17.57)	2.07 (1.15,3.74)	1.76 (0.88,3.53)	0.11
**Time to reach health facility**					
< 1 h	217 (65.75)	102 (61.82)	0.72 (0.42,1.23)	0.59 (0.317,1.11)	0.1
1-2 h	70 (21.21)	35 (21.21)	0.77 (0.41,1.43)	0.8 (0.39,1.64)	0.54
> 2 h	43 (13.03)	28 (16.97)	1	1	
**History of substance abuse**					
Yes	49 (14.85)	37 (22.42)	1.66 (1.03,2.67)	1.37 (0.78,2.42)	0.27
No	281 (85.15)	128 (77.58)	1		
**Physical abuse**					
Yes	23 (6.97)	21 (12.73)	1.95 (1.04,3.63)	2.13 (1.05,4.32)	0.036**
No	307 (93.03)	144 (87.27)	1	1	
**Experienced stress**					
Yes	76 (23.03)	52 (31.52)	1.54 (1.01,2.33)	0.76 (0.42,1.36)	0.35
No	254 (76.97)	113 (68.48)	1	1	

*COR* Crude Odd Ratio, *AOR* Adjusted Odd Ratio, *CI* Confidence Interval

*shows significant at *P*-value < 0.05

## Discussion

Globally, adverse birth outcomes remained the major public health problems in the world, particularly in developing countries where the accessibility and utilization of health care facilities are limited. This study was conducted to identify determinants of adverse birth outcomes among mothers who gave birth at public hospitals of Wollega zones. In this study, Antenatal care attendance, premature rupture of the membrane, being anemic, pregnancy-induced hypertension, dietary supplementation, and physical abuse were significantly associated with the development of the adverse birth outcome.

In this study, the odds of developing adverse birth outcomes were 3.92 times higher in mothers who have one-time ANC attendance than mothers who attended four and above times (AOR = 3.92: 95% CI; 1.86, 8.2). This finding is congruent with the results from Gonder [14], Hawassa [13], Dessie [15], North Wollo [17], and Tigray [27], in which adverse birth outcome is higher among mothers with limited ANC visit. This might be due to the fact that counseling given during the ANC follow up helps the mothers to get information on diet, danger signs of pregnancy, and pregnancy-related complication. It is known that ANC visits during pregnancy help to monitor the wellbeing of the fetus. The lack of adequate ANC visits during pregnancy decreases the chance of identifying risks of adverse birth outcomes and providing appropriate interventions for its prevention. Women should access to information related to nutrition and danger signs of pregnancy and ANC follow up will also help a woman seek early treatment for pregnancy related problems. Therefore, the regional health bureau should give more attention on expanding antenatal care especially in the rural areas, and create awareness about family planning methods for all women of reproductive age group both in rural and urban setting.

The odds of developing adverse birth outcomes were 2 times higher among anemic mothers than their counterparts (AOR = 2.0: 95% CI; 1.16,3.44). This finding is consistent with the study done in Dessie [15], Tigray [27], India [31]. Anemia, which is common during pregnancy, particularly in developing countries, highly contributed to the adverse birth outcome. Anemia may lead to decreased blood flow to the placenta and results in preterm labor which in turn results in preterm birth and other adverse birth outcomes. Also, anemia may cause hypoxia which can induce fetal stress, which stimulates the production of the corticotrophin-releasing hormone (CRH) leading to preterm labor. Iron deficiency may also increase the risk of maternal infections which can again stimulate the production of CRH predisposing to preterm birth. Therefore, evaluation of hemoglobin level and dietary supplementation is highly recommended throughout pregnancy time.

Premature rupture of the membrane (PROM) is one of the significant factors associated with adverse birth outcomes [32]. The finding of the study showed that the odds of developing adverse birth outcomes were 2.83 times higher among mothers who developed PROM than mothers who do not develop (AOR = 2.83: 95% CI; 1.72,4.64). The reason for this might be due to the fact that premature rupture of the membrane leads to umbilical cord prolapse, placental abruption, and fetal distress. PROM can lead to uterine contraction as amniotic fluid contains prostaglandin which in turn may result in adverse birth outcomes. Besides, PROM elevates fetal plasma interleukin-6 which may trigger preterm labor and leads to preterm delivery. PROM is also associated with lower latency from membrane rupture until delivery and causes around 25–30% of all preterm deliveries.

The hypertensive disorder is one of the most common problems encountered during pregnancy and contribute to the adverse birth outcome. This study revealed that mothers who developed pregnancy-induced hypertension were 2.3 times higher odds of developing adverse birth outcomes than their counterparts (AOR = 2.3:95% CI;1.4,3.85). This finding is supported by a study done in Suhul hospital Shire [33] and Gonder [14] in which pregnancy-induced hypertension is a high-risk factor for adverse birth outcomes. Another prospective cohort study done in Ethiopia indicated that a higher number of adverse prenatal outcomes occurred among pregnancy-induced hypertensive women [34]. This might be due to the fact that hypertension which is occurred during pregnancy increase the risk of fetus intrauterine growth restriction which further contributed to adverse birth outcomes. Hypertensive disorder can cause vascular damage to the placenta causing abruption placenta. When the blood pressure becomes uncontrollable, the quickest means of emptying the uterus become the choice accounting for the preterm delivery. In addition, elevated blood pressure in pregnancy compromises perfusion to the fetus and has a medical risk of cardiovascular complications for the mother. So, early screening of hypertensive disorders of pregnancy at the time of antenatal follow up is very important, and the health care professionals especially midwives should emphasize on this issue.

The study results also revealed that the odds of developing adverse birth outcomes were 2.47 times higher among mothers who didn’t get dietary supplementation than their counterparts (AOR = 2.47:95% CI;1.6,3.82). A systematic review study indicated that nutrients have a profitable effect on reducing adverse birth outcomes [35]. Another study conducted in Iran [36], also concluded as dietary supplementation reduced maternal and birth complications. The reason for this might be due to the fact that supplementation of diet during pregnancy helps the mothers and fetuses to get adequate nutrient which contributes to normal growth and prevent complications. When the mother’s nutritional status is poor; they will be prone to chronic infection which may lead to the activation of the maternal-fetal immune system causing preterm labor. So, health care personnel working on antenatal care should appropriately counsel the pregnant mother on the importance of dietary supplementation for the health of the mother and fetus.

The study findings also indicated that mothers who experienced physical abuse were 2.13 times more likely to develop adverse birth outcomes than mothers who do not exposed to physical abuse (AOR = 2.13: 95% CI;1.05,4.32). This finding is supported by the study done in Tigray [37] and Hosanna Town [38], in which physical abuse and violence were associated with adverse birth outcomes. The reason for this might be physical abuse during pregnancy may lead to physical injury to the fetus, abortion, and premature rupture of the membrane and finally may cause adverse birth outcomes.

## Conclusions

In this study, low ANC visits, being anemic, premature rupture of membrane, pregnancy-induced hypertension, not getting dietary supplementation, and physical abuse were determinants of adverse birth outcomes. The clinicians should play a pivotal role to improve ANC follow up, counsel, and supplement recommended diets and minimize violence and abuse during pregnancy.

## Supplementary Information

**Additional file 1.**

## Data Availability

All data used in this study are available from the corresponding author on reasonable request.

## Abbreviations

ANC: Antenatal care
AOR: Adjusted odds ratio
BMI: Body mass index
CI: Confidence interval
COR: Crude odd ratio
CSA: Central statistical agency
HIV: Human immunodeficiency virus
KG: Kilogram
LMP: Last menstrual period
PIHTN: Pregnancy-induced hypertension
PROM: Premature rupture of membrane
SPSS: Statistical package for social sciences
SVD: Spontaneous vaginal delivery
VIF: Variance inflation factor
