# Supplementary material for: Determinants of adverse birth outcomes among women delivered in public hospitals of Ethiopia, 2020
Source: Arch Public Health. 2022 Jan 4;80:12. doi: 10.1186/s13690-021-00776-0 (PMC8728986; doi:10.1186/s13690-021-00776-0)
Supplement: Supplementary file 1 — Additional file 1. [file 13690_2021_776_MOESM1_ESM.docx]

**Wollega university**

**Institute of health sciences**

**Consent form**

This questionnaire is prepared by Wollega university, School of nursing & midwifery research team to conduct the study on “**Determinants of adverse birth outcomes among women delivered in public hospitals of Western Ethiopia, 2020: Unmatched case-control study”.** You have been included randomly to this study and the information you will provide will neither be disclosed to the third party, nor be used for another purpose and will be rather kept confidential. The information collected will not be linked to you and you don’t need to tell your name. You have full right to participate, reject or not to answer all or part of the questions. However, your participation in giving the right information is quite important for our study.

Do you agree to participate?

Yes Continue your interview

No Thank him or her

Name of interviewer/data collector

Signature

Crosschecked by the supervisor A/yes B/No Signature

**Questionnaires**

**Part I: Sociodemographic characteristics of the mothers**

1. What is your age in years? _____________
2. What is your age at first marriage in years? ___________
3. What is your Ethnicity?
4. Oromo B. Amhara C. Gurage D. Tigre E. Others
5. What is your religion?
6. Protestant B. Orthodox C. Catholic D. Muslim E. Others
7. What is your current marital status?
8. Married B. Single C. Divorced D. Widowed
9. What is your highest educational status?
10. Unable to read and write C. Completed grade 9-12
11. Completed grade 1-8 D. Diploma and above
12. What is your current occupation?
13. Government employee D. Farmer E. Others
14. Private employee E. Merchant
15. What is your husband’s current occupation?
16. Government employee C. Merchant E. Others
17. Private employee D. Farmer
18. What is your residence?
19. Urban B. Rural
20. What is your family size in number? ______________
21. What is your family’s average monthly income in birr? ______________
22. Time to reach nearby health facility in hour ___________

**Part II: Obstetrics related characteristics of the mothers**

1. How many times you gave birth including the current birth?

A. 1^st^ pregnancy

B. 2 times

C. 3 times

D. 4 times

E. > 4 times

2. What is the duration between the current & previous childbirth in years?

A. < 2 years

B. ≥ 2 years

3. Did you or your partner use family planning before this current pregnancy?

A. Yes B. No

4. Did you plan this pregnancy?

A. Yes B. No

5. What was your status of antenatal care attendance for the current pregnancy?

A. Not attended at all

B. 1 time

C. 2 times

D. 3 times

E. ≥ 4 times

6. What is the mode of delivery for the current pregnancy?

A. SVD C. CS E. Destructive delivery

B. Forceps D. Vacuum delivery

7. Have you had history of previous preterm birth?

A. Yes B. No

8. Have you experienced premature rupture of membrane in this current pregnancy?

A. Yes B. No

9. How is your current labor started?

A. Spontaneous B. Induced

10. Have you had history of abortion?

A. Yes B. No

11. What is your current number of child born?

A. Singleton B. Multiple

**Part III: Medical history related characteristics of mothers**

1. Have you had a history of diabetes mellitus during the current pregnancy?
2. Yes B. No
3. Have you had a history of cardiac disease during the current pregnancy?
4. Yes B. No
5. What is your HIV/AIDS status?
6. Positive B. Negative C. Unknown
7. Have you experienced anemia during the current pregnancy?
8. Yes B. No
9. Have you experienced malaria during the current pregnancy?
10. Yes B. No
11. Have you a history of Sexually transmitted diseases during this current pregnancy?
12. Yes B. No
13. Have you developed hypertensive disorders during the current pregnancy?
14. Yes B. No

**Part IV: Social, behavioral and nutritional related characteristics of mothers**

1. Have you had history of substance use?
2. Yes B. No
3. Did you get dietary supplementation during this current pregnancy?
4. Yes B. No
5. Maternal MUAC measurement in centimeters ______________
6. Did you get family/social support during the current pregnancy?
7. Yes B. No
8. Have you experienced physical abuse during this pregnancy?
9. Yes B. No
10. Have you ever used traditional medicine?
11. Yes B. No
12. Have you experienced any type of stress during the current pregnancy?
13. Yes B. No

**Part V: Stress measurement**

PERCEIVED STRESS SCALE: The questions in this scale ask you about your feelings and thoughts during the last month. In each case, you will be asked to indicate by circling how often you felt or thought a certain way.

0 = Never 1 = Almost Never 2 = Sometimes 3 = Fairly Often 4 = Very Often

1. In the last month, how often have you been upset because of something that happened unexpectedly?

2. In the last month, how often have you felt that you were unable to control the important things in your life?

3. In the last month, how often have you felt nervous and “stressed”?

4. In the last month, how often have you felt confident about your ability to handle your personal problems?

5. In the last month, how often have you felt that things were going your way?

6. In the last month, how often have you found that you could not cope with all the things that you had to do?

7. In the last month, how often have you been able to control irritations in your life?

8. In the last month, how often have you felt that you were on top of things?

9. In the last month, how often have you been angered because of things that were outside of your control?

10. In the last month, how often have you felt difficulties were piling up so high that you could not overcome them?

**Part VI: Danger signs of pregnancy**

Have you developed any of the following danger sign of pregnancy?

(1) severe vaginal bleeding

(2) convulsions

(3) severe headache with blurred vision

(4) severe abdominal pain

(5) too weak to get out of bed

(6) fast or difficulty in breathing

(7) reduced fetal movement

(8) fever

(9) swelling of the fingers, face, and legs.

**Part VII: Birth outcome assessment (To be filled from patient cards & measurement)**

1. Preterm birth (Before 37 completed weeks of gestation)?
2. Yes B. No
3. Low birth weight (Birth weight below 2500 g)?
4. Yes B. No
5. Macrosomia (Birth-weight over 4000 g)?
6. Yes B. No
7. Still birth (Infant delivered with no sign of life)?
8. Yes B. No
9. Birth defect/congenital abnormality (structural changes in one or more parts of the body)?
10. Yes B. No
11. Neonatal death (death of infant or neonates within 28 days of life)?
12. Yes B. No
13. Small for gestational age (birth weight below the 10th percentile for the gestational age)?
14. Yes B. No
15. Number of ABO observed in a new birth?
16. No ABO observed
17. Only 1 ABO observed
18. 2 or more ABO observed
19. Over all ABO related to the child (met at least one of the above conditions)?
20. No B. Yes

THANK YOU VERY MUCH!
